# Prognostic Indicators for Ebola Patient Survival

**DOI:** 10.3201/eid2202.151250

**Published:** 2016-02

**Authors:** Samuel J. Crowe, Matthew J. Maenner, Solomon Kuah, Bobbie Rae Erickson, Megan Coffee, Barbara Knust, John Klena, Joyce Foday, Darren Hertz, Veerle Hermans, Jay Achar, Grazia M. Caleo, Michel Van Herp, César G. Albariño, Brian Amman, Alison Jane Basile, Scott Bearden, Jessica A. Belser, Eric Bergeron, Dianna Blau, Aaron C. Brault, Shelley Campbell, Mike Flint, Aridth Gibbons, Christin Goodman, Laura McMullan, Christopher Paddock, Brandy Russell, Johanna S. Salzer, Angela Sanchez, Tara Sealy, David Wang, Gbessay Saffa, Alhajie Turay, Stuart T. Nichol, Jonathan S. Towner

**Affiliations:** Centers for Disease Control and Prevention, Atlanta, Georgia, USA (S.J. Crowe, M.J. Maenner, B.R. Erickson, B. Knust, J. Klena, C.G. Albariño, B. Amman, J.A. Belser, E. Bergeron, D. Blau, S. Campbell, M. Flint, A. Gibbons, L. McMullan, C. Paddock, J.S. Salzer, A. Sanchez, T. Sealy, D. Wang, S.T. Nichol, J.S. Towner);; International Rescue Committee, New York, New York, USA (S. Kuah, M. Coffee, D. Hertz);; Ministry of Health and Sanitation, Bo Town, Sierra Leone (J. Foday, G. Saffa, A. Turay);; Médecins Sans Frontières, Brussels, Belgium (V. Hermans, M. Van Herp); Médecins Sans Frontières, London, UK (J. Achar, G.M. Caleo);; Centers for Disease Control and Prevention, Fort Collins, Colorado, USA (A.J. Basile, S. Bearden, A.C. Brault, C. Goodman, B. Russell)

**Keywords:** Ebola virus disease, hemorrhagic fever, survival, prognosis, Sierra Leone, viruses, zoonoses, Ebola, Ebola virus

## Abstract

To determine whether 2 readily available indicators predicted survival among patients with Ebola virus disease in Sierra Leone, we evaluated information for 216 of the 227 patients in Bo District during a 4-month period. The indicators were time from symptom onset to healthcare facility admission and quantitative real-time reverse transcription PCR cycle threshold (C_t_), a surrogate for viral load, in first Ebola virus–positive blood sample tested. Of these patients, 151 were alive when detected and had reported healthcare facility admission dates and C_t_ values available. Time from symptom onset to healthcare facility admission was not associated with survival, but viral load in the first Ebola virus–positive blood sample was inversely associated with survival: 52 (87%) of 60 patients with a C_t_ of >24 survived and 20 (22%) of 91 with a C_t_ of <24 survived. C_t_ values may be useful for clinicians making treatment decisions or managing patient or family expectations.

## Abstract 2

Odds of survival were greatest when first Ebola virus–positive blood sample collected had low viral load.

The epidemic of Ebola virus (*Zaire ebolavirus*) disease (EVD) in West Africa began in eastern Guinea in December 2013 (*1*) and quickly spread into Liberia and Sierra Leone, eventually overwhelming the fragile healthcare infrastructures in these countries (*2*). During the peak of the epidemic, many healthcare facilities were quickly filled beyond capacity, which often forced clinicians to make difficult decisions about how to triage patients and how to manage patient and family expectations regarding probable outcomes. Reliable prognostic indicators available at the time of patient admission could help clinicians make these decisions.

We therefore assessed the reliability of 2 potential prognostic indicators: 1) the total elapsed time from reported symptom onset to healthcare facility admission and 2) cycle threshold (C_t_), which can serve as an approximation of viral load, at the time of EVD diagnosis. Early treatment, which is made possible by early admission, is thought to improve chances of survival (*3*–*5*), but there is little supporting empirical evidence. Analyses of EVD patients in Ebola treatment units (ETUs) have shown that C_t_ values predict outcomes (*6**–**8*), but these studies do not account for those who died before ETU admission. By using onset-to-outcome data for all identified EVD patients during a 4-month period in Bo District, Sierra Leone, we explored the extent to which these indicators predicted outcome.

## Methods

### Population

Bo District is 1 of 14 districts in Sierra Leone and is located in the southern part of the country. Bo Town is the district capital, a major urban center, and the second largest city in Sierra Leone. Bo District consists of 15 chiefdoms, many of which are in rural areas, and includes ≈1,000 villages.

When patients suspected to have EVD were identified in Bo District, they were taken to the Ebola isolation unit in the government hospital in Bo Town. After a patient was admitted, blood was collected for Ebola virus testing and supportive care was provided (included oral rehydration therapy, paracetamol for fever, and sometimes presumptive care for other diseases such as malaria). During the first 2 months of the study period, the isolation unit did not consistently provide supportive care, but during the second 2 months, after the unit was reorganized by a new management team, the unit did provide such care. Throughout the 4-month study period, patient blood samples were transferred from this isolation unit to the field diagnostic laboratory, located a few kilometers away.

Patients with confirmed EVD were transferred to an ETU managed by Médecins Sans Frontières. In the ETU, located in the same compound as the field laboratory, the patients received care for EVD (fluid replacement, fever and pain medication, and antidiarrheal and antiemetic drugs), as well as presumptive care for other diseases, nutritional support, and psychosocial counseling (*9*). Occasionally, patients sought care first at the ETU and were admitted directly into that facility. Data for evaluating the treatment provided at either of these facilities were not available.

### Data Sources

We collected data for all identified persons from Bo District who had confirmed EVD and a symptom onset date from September 12, 2014, through January 7, 2015. To have the most complete and accurate data, we relied on multiple sources: 1) demographic information and symptom onset dates from the case investigation forms; 2) admission dates and death reports from the government hospital isolation unit; 3) ETU admission dates and patient outcomes (survival or death); 4) EVD diagnostic test results; and 5) confirmation of deaths from the district burial team, which buried the bodies of deceased EVD-positive patients.

These sources routinely reported this information to the Bo District surveillance team, which maintained a database by using the Epi Info Viral Hemorrhagic Fever application (https://epiinfovhf.codeplex.com/). In the event of missing or conflicting information, we requested verification or additional information from the original sources. For each infected person, we compiled symptom onset date; healthcare facility admission dates; outcome type and date; patient age, sex, and place of residence; and laboratory test results. To ensure a complete linkage and to identify persons with duplicate records, we reviewed all information for errors.

This assessment was considered to be a nonresearch public health response activity and thus did not undergo institutional review board review. Because this secondary analysis used only information that had already been collected for public health surveillance and clinical management purposes, informed consent was not obtained.

### Measurement of Time from Symptom Onset to Admission

Time from symptom onset to healthcare facility admission was calculated by subtracting the reported symptom onset date from the admission date and was recorded in days. We used as many as 3 recorded admission dates: dates of admission to the local clinic where EVD was first suspected, to the isolation unit, and to the ETU. To better examine the changing circumstances during the epidemic, we created 3 groups of patients according to the type of facility where they were admitted: 1) all EVD patients admitted to any healthcare facility (primary cohort), 2) only patients admitted to the ETU (ETU subgroup), and 3) patients admitted to the isolation unit during the last 2 months of the assessment (November 16–January 10) when patients were consistently receiving care in the unit (final 2 months subgroup).

### Measurement of C_t_

The field laboratory, operated by the US Centers for Disease Control and Prevention (CDC), tested persons suspected of having EVD by using nucleoprotein (NP) and viral protein (VP) 40 quantitative real-time reverse transcription PCRs and a β-2-microglobin control. These tests detect Ebola viral RNA in blood specimens (*10*,*11*). C_t_ is defined as the number of cycles of RNA replication that have occurred when the Ebola virus–specific RNA signal is detected. A total of 40 cycles of replication are run for a given specimen; if no RNA signal is detected and the β-2-microglobin control result is positive, the test result is negative. Therefore, the lower the C_t_ for a positive specimen, the higher the relative quantity of virus.

For this analysis, only the C_t_ values from the VP40 assay were used because this assay was slightly more sensitive than the NP assay (i.e., the VP40 detected positive cases that the NP did not). The association between C_t_ and 50% tissue culture infective doses per milliliter (TCID_50_/mL) is provided in the CDC document Ebola Virus VP40 Real-Time RT-PCR Assay (*11*). Each 3-point decrease in C_t_ was associated with an ≈10-fold increase in Ebola viral load; a C_t_ of 39 corresponded to ≈40 TCID_50_/mL and a C_t_ of 19 corresponded to ≈40 million TCID_50_/mL (*11*). Standard curves were not determined for each run; therefore, the viral load for each patient was an approximation. Samples with a C_t_ of <40 were classified as EVD-positive. If a person was tested within 72 hours of symptom onset and the test result was negative, that person was generally retested to confirm the negative result (*12*). Confirmatory tests for deceased persons were performed by using body fluids collected from oral swab samples, whereas testing of live patients was performed on whole blood, serum, or plasma.

### Statistical Analyses

Because it is unknown whether C_t_ values from swab samples and blood tests yield comparable results, we excluded from the primary cohort and the 2 subgroups all infected persons for whom EVD was detected after death (and thus tested by oral swab sampling). We also excluded patients for whom C_t_ values or admission dates were not available.

We calculated the case-fatality proportion for all patients in Bo District for whom outcome was known, who were admitted to a healthcare facility, and who were admitted to the ETU. We stratified the primary cohort and the 2 subgroups by patient outcome and described those who survived and those who died in terms of sex, average age, average C_t_ at first test, and average number of days from symptom onset to healthcare facility admission.

We examined the distribution of C_t_ values for the Ebola patients and created a scatterplot with a LOESS (locally weighted scatterplot smoothing) curve to serve as a graphical representation of patient survival by C_t_. We did this with the LOESS function in R (https://www.r-project.org/) by using the default span of 0.75 and degree of 2. We determined the C_t_ that was most accurate when used as a dichotomous predictor for survival. We also categorized C_t_ into 3 levels according to visual inspection of the relationship between C_t_ and survival.

We ran unadjusted logistic regression analyses in R to determine if the following covariates are associated with patient survival: sex, age (continuous and categorical variable), C_t_ (continuous and categorical variable), and days from symptom onset to admission to a healthcare facility (any facility, ETU, isolation ward during last 2 months of the assessment). We ran 3 multivariable logistic regression models in R (1 for each group) that included sex, age (continuous variable), C_t_ (categorical variable), and days from reported symptom onset to admission to a healthcare facility. We used the Pearson correlation coefficient to examine collinearity between time from symptom onset to healthcare facility admission and C_t_.

## Results

**Figure 1:**
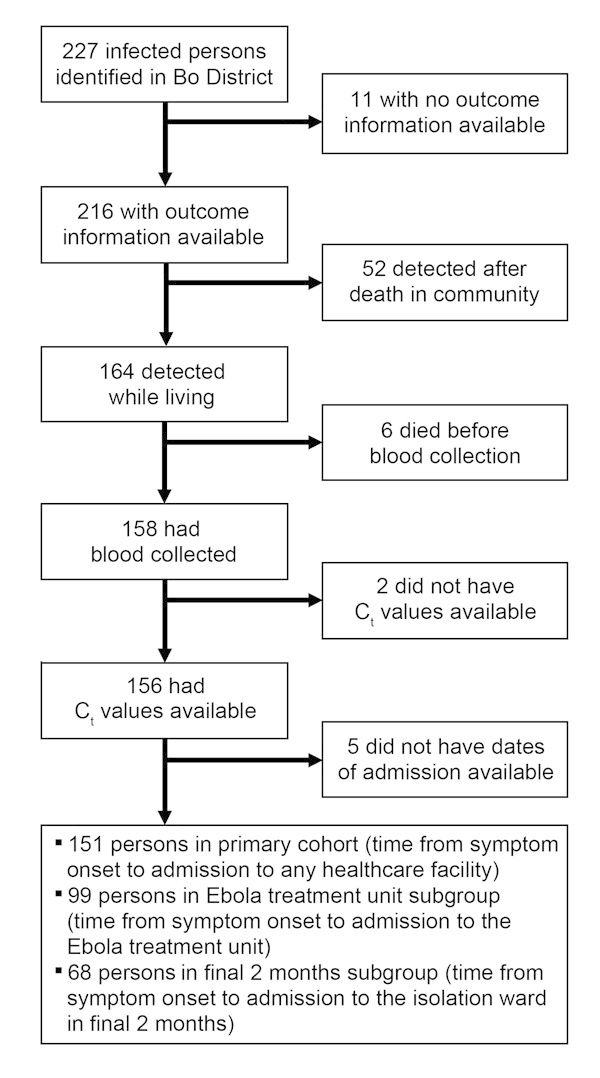
Classification of patients with Ebola virus disease into study groups, Bo District, Sierra Leone, September 2014–January 2015.

During our study period, the surveillance system identified 227 Bo District residents with EVD. Outcome (death or recovery) could be confirmed for 216 patients, but outcome information was missing for 11. Of the 216 patients with outcome data, 164 were detected and admitted to a healthcare facility, but the other 52 died in the community before being detected. Of the 164 patients, 6 died before blood could be collected for confirmatory testing, C_t_ values were missing for 2, and admission dates were missing for 5. The primary cohort comprised the remaining 151 patients. Although 123 patients were admitted to the ETU, admission dates were known for only 99; the ETU subgroup comprised these 99 patients. Dates of admission to the isolation ward during the final 2 months of the study period (when treatment was consistently provided) were known for another 68 patients; the final 2 months subgroup comprised these 68 patients (Figure 1).

Outcome was known for 216 persons, of whom 142 (66%) died. Among the 164 persons admitted to a healthcare facility, 90 (55%) died. Among the 123 persons admitted to the ETU, 49 (40%) died (including 8 admitted directly to the ETU, 3 [38%] of whom died). All survivors were ultimately discharged from the ETU.

Approximately half of the patients in the primary cohort (52%), the ETU subgroup (49%), and the final 2 months subgroup (50%) died. Of the 151 patients in the primary cohort, 90 (60%) were female; of these, 47 (52%) died. The 2 subgroups had similar proportions. The mean age (in years) of survivors in each of the 3 groups was low- to mid-20s, and the mean age for those who died was low- to mid-30s. The mean C_t_ for the survivors in the 3 groups was in the upper 20s and the mean C_t_ for the deceased was in the low 20s. The mean number of days from symptom onset to healthcare facility admission was nearly same for those who survived and those who died (Table 1).

**Table 1:** Characteristics of patients with confirmed Ebola virus disease, Bo District, Sierra Leone, September 2014–January 2015

Characteristic	Primary cohort, n = 151			Ebola treatment unit subgroup, n = 99			Final 2 months subgroup, n = 68	
	Survived	Died		Survived	Died		Survived	Died
No. patients	72	79		50	49		34	34
No. female	43	47		31	32		16	23
Mean age, y	24.1	31.7		24.8	30.1		20.2	33.0
Mean cycle threshold	27.9	20.5		27.9	21.2		26.8	21.4
Mean time from symptom onset to admission, d	3.5	3.7		6.0	5.6		3.4	3.5

**Figure 2:**
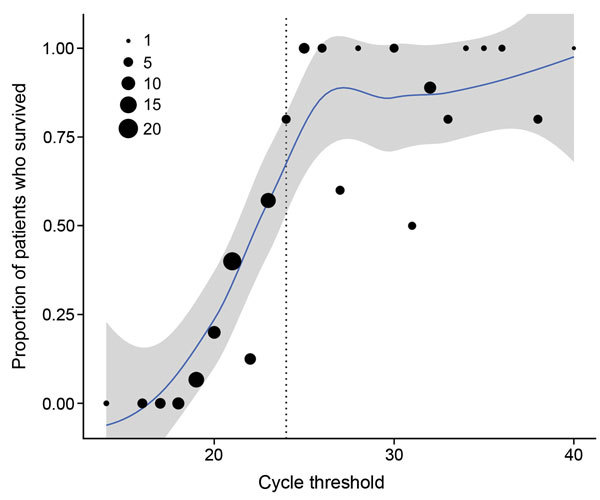
Percent survival among 151 patients in the Ebola virus disease (EVD) primary cohort by cycle threshold (C_t_) rounded to nearest integer, Bo District, Sierra Leone, September 2014–January 2015. Locally weighted smoothing line and 95% uncertainty intervals added to illustrate trend. The area of each dot is scaled to represent the number of confirmed EVD cases, by C_t_. The trend line suggests a sharp increase in survival for patients with C_t_ values in the mid-20s (dotted line).

The average chance of survival among patients in the primary cohort showed a sharp increase for those with C_t_ values in the low- to mid-20s (Figure 2). Of note, 52 (87%) of 60 patients for whom C_t_ was >24 survived, whereas only 20 (22%) of 91 with a C_t_ of <24 survived. Of all 72 survivors, C_t_ was >24 for 52 (72%); of the 79 who died, C_t_ was <24 for 71 (90%).

Unadjusted logistic regression models indicate that C_t_ —as both a continuous and a categorical variable—is strongly associated with survival (Table 2). Among those in the primary cohort, older age in years (as a continuous variable) also was inversely associated with survival (odds ratio [OR] 0.97, 95% CI 0.95–0.99), meaning that younger patients were more likely to survive. Male patients had nearly the same odds of surviving as did female patients (OR 0.99, 95% CI 0.52–1.90). Also among those in the primary cohort, symptom onset to admission to any healthcare facility was not associated with survival (OR 0.97, 95% CI 0.87–1.08) (Table 2).

**Table 2:** Logistic regression models assessing association of patient sex, age, C_t_, and time from Ebola virus disease symptom onset to healthcare facility admission with patient survival, Bo District, Sierra Leone, September 2014–January 2015*

Cohort	No. patients	Unadjusted			Adjusted†	
		OR for survival (95% CI)	p value		OR for survival (95% CI)	p value
Primary cohort						
Male, vs. female	151	0.99 (0.52–1.90)	0.98		0.96 (0.38–2.44)	0.94
Age, y, increasing, continuous	151	0.97 (0.95–0.99)	0.009		0.97 (0.94–0.99)	0.01
Age ≥20 y, vs. <20 y	151	0.54 (0.27–1.06)	0.076			
C_t_, decreasing, continuous	151	0.73 (0.65–0.80)	<0.001			
C_t_ <20, vs. >24	151	0.0044 (0.0002–0.0245)	<0.001		0.003 (0.001–0.018)	<0.001
C_t_ 20–24, vs. >24	151	0.12 (0.04–0.28)	<0.001		0.086 (0.028–0.22)	<0.001
C_t_ <24, vs >24	151	0.04 (0.02–0.10)	<0.001			
Days from symptom onset to admission to any healthcare facility, increasing, continuous	151	0.97 (0.87–1.08)	0.59		0.88 (0.76–1.02)	0.089
ETU subgroup						
Days from symptom onset to admission to ETU, increasing, continuous	99	0.94 (0.83–1.07)	0.37		0.88 (0.74–1.03)	0.11
Final 2 months subgroup						
Days from symptom onset to admission to isolation ward, increasing, continuous	68	0.98 (0.79–1.20)	0.84		0.85 (0.64–1.11)	0.23

*C_t_, cycle threshold; ETU, Ebola treatment unit; OR, odds ratio. †Adjusted for sex, age (continuous), and C_t_ (<20, 20–24, >24).

**Figure 3:**
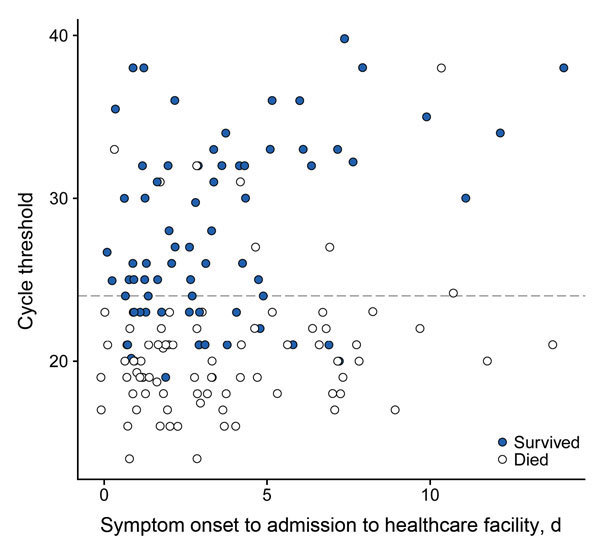
Scatterplot of outcome by cycle threshold (C_t_) at time of first Ebola virus–positive test result and time to admission at any healthcare facility (primary cohort, n = 151), Bo District, Sierra Leone, September 2014–January 2015. Each circle represents an infected person. The dashed line indicates the classification threshold of the C_t_ value of 24. Observations are slightly horizontally jittered to reduce overplotting.

In the adjusted analysis of the primary cohort, the association of C_t_ with survival was not attenuated; the OR point estimates were more extreme in all parameterizations of C_t_. The association found in the unadjusted models between age and survival, and the lack of association between patient sex and survival, remained virtually the same in the adjusted analysis. After adjustment of the analysis, time from symptom onset to admission was not significantly associated with survival for those in the primary cohort (OR 0.88, 95% CI 0.76–1.02). Analysis results for the 2 subgroups were similar (ETU subgroup OR 0.88, 95% CI 0.74–1.03; final 2 months subgroup OR 0.85, 95% CI 0.64–1.11) (Table 2) and produced very similar ORs for the other covariates (data not shown). This tenuous association between time from symptom onset to admission and survival may be driven by a small number of patients with long times from symptom onset to admission; when the primary cohort is restricted to patients for whom reported time of symptom onset to admission was <10 days (145 of 151 patients), the magnitude of the association was greatly diminished (adjusted OR 0.97, p = 0.82). The Pearson correlation test between the time from symptom onset to admission to a healthcare facility and C_t_ yielded a small but statistically significant relationship (r = 0.19, p = 0.01). C_t_ values were slightly higher for patients for whom time from symptom onset to admission was longer than for those for whom this time was shorter (Figure 3).

## Discussion

The overall observed case-fatality proportion of 66% in this assessment is higher than some other case-fatality estimates for this epidemic but less than those reported for many previous outbreaks of EVD (*13*–*15*). As of November 18, 2015, the World Health Organization reported a 41% (3,589/8,704) case-fatality proportion in Sierra Leone (*16*), and an ETU in Freetown, the country’s capital city, reported a mortality rate among its patients of 31% (*17*). Although the higher case-fatality proportion for Bo District could reflect specific circumstances (such as differences in severity of illness, access to care, or patient care-seeking behavior), it could also reflect more complete outcome ascertainment. For instance, the case-fatality proportion was lower (55%) when community deaths were excluded or when only those who survived long enough to be admitted to the ETU (40%) were considered; these proportions are more in line with some estimates based on ETU patients only (*6*,*17*). Another analysis, from a subset of patients in Sierra Leone for whom outcomes were known, estimated a mortality rate of 69%, similar to that found in this study (*18*). Including deaths from community and healthcare facility sources could increase the estimated lethality of EVD in Sierra Leone and perhaps more generally in West Africa (*19*).

Community members were commonly told that patients who receive care for EVD soon after symptom onset have a better chance of survival (*3*,*4*), in part because severe diarrhea is a prominent feature of the disease (*20*,*21*). Accordingly, the sooner EVD patients receive care that counteracts the deleterious effects of substantial fluid loss, the less likely is development of hypovolemia and multiple organ failure (*22*,*23*). This position is both intuitive and biologically plausible, so we were surprised that the association between survival and time from symptom onset to admission to a healthcare facility did not reach statistical significance. Although seemingly counterintuitive, our finding is similar to that of a recent analysis conducted in another area of Sierra Leone (*6*). One explanation for these findings is that the average time to admission (3.5 days) was too long to demonstrate an association with survival. Many patients were far along in the course of the disease by the time they received supportive care; 43% died before reaching the ETU. The goal of reducing the time to receipt of care is laudable but could be challenging in a setting like West Africa, where many villages are located far from healthcare facilities. More likely, improving the availability of more sophisticated supportive care or developing advanced therapies, such antiviral drugs, will be needed to improve outcomes, particularly among the sickest patients. However, earlier detection and treatment remains a priority for every Ebola response because quicker isolation of EVD patients probably reduces transmission (*24*–*27*).

Similar to the results of some analyses of ETU patients (*6*–*8*), we found that C_t_ of the first Ebola virus–positive sample was strongly associated with survival. Infected persons with lower C_t_ values (thus higher viral loads) at time of detection were more likely to die than were those with higher C_t_ values. This finding was consistent across patients regardless of time from symptom onset to admission to any healthcare facility (Figure 3), suggesting variability in the severity and course of the illness. The differences among patients can be stark: a patient with mild illness (and lower viral load) might not notice the initial onset of symptoms, wait longer for treatment, and still be more likely to survive than someone with rapid and severe illness onset who is immediately sent to the ETU.

To date, quantitative real-time reverse transcription PCR has been used in the Ebola response to distinguish EVD cases from non-EVD cases and to determine when a convalescing patient can be released from an ETU. However, evidence that C_t_ might also be useful as a prognostic tool is increasing. This finding was first reported >10 years ago after an outbreak of Sudan virus infection (*28*). Since then, EVD analyses supporting this position have been conducted in Sierra Leone and Guinea by using data from a few healthcare facilities in each country (*6*–*8*). Our assessment supports and adds to this body of evidence as a population-based sample that includes patients who died before admittance to an ETU.

Using C_t_ as a prognostic indicator could have several benefits for clinicians. First, it could guide patient triage and help clinical staff determine the best treatment course for the gravely ill, particularly when intravenous fluids or advanced supportive therapies are in short supply. Such therapies might help improve patient outcomes when used on those who have higher viral loads at the time of initial care and those who are more ill than their peers. Second, if a patient has mild symptoms but a high viral load, clinicians can be prepared for the patient’s condition to deteriorate quickly. Third, healthcare staff also can use this information to manage patient and family expectations regarding probable outcomes.

Limitations of this study include probable misreporting of patient age and symptom onset date, as well as the relatively small number of cases. Although we included all known deaths among persons with EVD to calculate the overall case-fatality proportion, the 58 persons who died before or shortly after detection were excluded from the analysis because comparable blood samples or reliable symptom onset dates were not available. This exclusion is potentially a source of bias if the time from symptom onset to death for these 58 persons is longer than the time from onset to admission for the remaining patients (Technical Appendix). In addition, although this study included all detected cases, it is unknown how many cases might not have been detected by the surveillance system. Outcomes of these undetected infected persons might have differed from those of detected persons, leading to an incorrect estimate of survival. The quality of care probably varied between facilities and between time periods, particularly in the isolation unit, which could have affected our findings. Furthermore, the C_t_ values used for this assessment are specific to the CDC laboratory equipment and VP40 assay used. Use of other laboratory equipment, procedures, or assays could yield different results, thus affecting the optimal C_t_. Standardization of equipment, procedures, and assays would facilitate the use of C_t_ as a prognostic indicator. Last, determining an optimal C_t_ for prognosis probably depends on specific patient characteristics and the effectiveness of care provided; the strength of the association between C_t_ and survival could be confounded by these or other unmeasured factors.

In summary, the case-fatality proportions found by this study were higher than estimates that do not include all deaths of known infected persons or that are limited to ETU patients only. A C_t_ of >24 in the first Ebola virus–positive sample was a strong predictor of survival among persons who were alive when detected by the surveillance system. In this population, the time from reported symptom onset to healthcare facility admission was not associated with survival. Additional studies are needed to validate these findings and to continue to explore how C_t_ values can be combined with other biomarkers (*29*,*30*) to provide insights into the effectiveness of treatment and prognosis.

**Technical Appendix.** Notes about persons excluded from analyses of prognostic indicators for Ebola virus disease survival, Sierra Leone.
