# Supplementary material for: Prognostic Indicators for Ebola Patient Survival
Source: Emerg Infect Dis. 2016 Feb;22(2):217–23. doi: 10.3201/eid2202.151250 (PMC4734506; doi:10.3201/eid2202.151250)
Supplement: Supplementary file 1 — Technical Appendix. Notes about persons excluded from analyses of prognostic indicators for Ebola virus disease survival, Sierra Leone. [file 15-1250-Techapp-s1.pdf]

Article DOI: http://dx.doi.org/10.3201/eid2202.151250

Prognostic Indicators for Ebola Patient Survival

Technical Appendix

Note on Persons Excluded from Prognostic Analyses

The analyses concerning the prognostic utility of Ct and time from onset to admission did not include individuals who died before blood could be drawn. Fifty-two of these people died in the community before they were detected, and 6 after detection but before they could have their blood drawn. For these 58 individuals, confirmatory testing was performed via oral swab, and Ct levels obtained from swabs are not directly comparable to those obtained from blood.

Of the 52 individuals who were dead when detected, 25 had dates of onset reported to the surveillance database. One person had a date of specimen collection (October 7) that preceded symptom onset date (October 12), so that person was excluded. For the remaining 24, we calculated the time from reported onset to specimen collection (at which point they were dead). For 6 of the 24, date of specimen collection was not available, so instead we used the date the sample was received by the laboratory (usually between 0–2 days after sample collection). Thus, duration from reported onset to date of specimen collection (or received by laboratory) will be an overestimate of the duration from reported onset to time of death.

This is the distribution of the time from onset to specimen collection/laboratory receipt of specimen for the 24 individuals with onset dates available:

Days	Frequency
0	1
2	8
3	3
4	7
5	1
7	2
9	1
10	1

For the above 24 bodies, the mean is 3.8 days.

Of the 6 individuals who were detected when alive but died before blood could be drawn, 5 had information for onset date (0, 2, 3, 4, and 14 days) with a mean of 4.6 days.

Together, the 29 individuals with information available have a mean of 3.9 days—very similar to the time from reported onset to initial health facility admission among the primary cohort (mean 3.5 days for those who survived and 3.7 days for those who died). These data suggest that the deceased did not, on average, go longer without care than the living. However, the data should be interpreted with a number of caveats in mind: the data were missing for half of the deceased individuals, time of specimen collection might overestimate time until death, and the ascertainment of symptom onset for a dead person might be less accurate than for living patients.
